# A Case Study of Environmental Injustice: The Failure in Flint

**DOI:** 10.3390/ijerph13100951

**Published:** 2016-09-27

**Authors:** Carla Campbell, Rachael Greenberg, Deepa Mankikar, Ronald D. Ross

**Affiliations:** 1 Department of Public Health Sciences, Room 408, College of Health Sciences, University of Texas at El Paso, 500 W. University Ave, El Paso, TX 79968, USA; 2 National Nurse-led Care Consortium (NNCC), Philadelphia, PA 19102, USA; rgreenberg@nncc.us; 3 Research and Evaluation Group, Public Health Management Corporation, Philadelphia, PA 19102, USA; dmankikar@phmc.org; 4 Occupational and Environmental Medicine Consultant, Las Cruces, NM 88001, USA; ronald.d.ross40.civ@mail.mil

**Keywords:** environmental justice, lead poisoning, water contamination, Flint water crisis

## Abstract

The failure by the city of Flint, Michigan to properly treat its municipal water system after a change in the source of water, has resulted in elevated lead levels in the city’s water and an increase in city children’s blood lead levels. Lead exposure in young children can lead to decrements in intelligence, development, behavior, attention and other neurological functions. This lack of ability to provide safe drinking water represents a failure to protect the public’s health at various governmental levels. This article describes how the tragedy happened, how low-income and minority populations are at particularly high risk for lead exposure and environmental injustice, and ways that we can move forward to prevent childhood lead exposure and lead poisoning, as well as prevent future Flint-like exposure events from occurring. Control of the manufacture and use of toxic chemicals to prevent adverse exposure to these substances is also discussed. Environmental injustice occurred throughout the Flint water contamination incident and there are lessons we can all learn from this debacle to move forward in promoting environmental justice.

## 1. Description of the Flint Water Crisis

At this point, most Americans have heard of the avoidable and abject failure of government on the local, state and federal level; environmental authorities; and water company officials to prevent the mass poisoning of hundreds of children and adults in Flint, Michigan from April 2014 to December 2015 [1,2,3]. One tends to imagine chemical poisoning as a victim dropping dead in a murder mystery, or the immediate casualties in an industrial accident or a chemical warfare attack. Unlike the release of methyl isocyanate gas in Bhopal, India in 1984 or the release of radiation with the radiation accident in Chernobyl, Ukraine in 1986, the poisoning of the population in Flint was an insidious one. People drinking the contaminated water would never have known they had elevated blood lead levels (BLLs) without specific medical testing for blood lead levels. In fact, if the water contamination had not been made public, most exposed children and their families would have never suspected they were being exposed over a 20-month period of time, and it would be expected that the water contamination and lead exposure would have continued up until today.

Lead can cause immediate acute poisoning but the subacute, moderate, long-term exposure impact of concern in Flint is more common, and much more insidious. Any resulting behavioral disturbance or loss of intellectual function would probably not been have linked by their physicians or families to lead poisoning, and instead accepted as something that had just occurred. Additionally, the adverse effects from this event may take years to surface as most negative health effects from low-level lead exposure develop slowly [4]. Hypertension and kidney damage may not present until long after the exposure. Any resulting behavioral disturbance or loss of intellectual function would probably have not been linked by their physicians or families to lead poisoning, and instead accepted as something that had just occurred.

The Flint disaster was due to the switch in water supply from Lake Huron to the Flint River, which was then not treated with an anti-corrosion chemical to prevent lead particles and solubilized lead from being released from the interior of water pipes, particularly those from lead service lines or those with lead solder. This water was known to be very corrosive, so corrosive that, in fact, it was not used by the nearby auto industry [2]. The General Motors plant switched to water from the neighboring Flint Township when General Motors noticed rust spots on newly machined parts [2]. This corrosive new water supply was then not treated with the anti-corrosion treatment, in noncompliance with the Environmental Protection Agency’s (EPA) Lead and Copper Rule, which calls for action when a water supply is found to be corrosive to prevent the potential release of metals from water service lines [5].

A national water expert, Dr. Marc Edwards, a professor of civil and environmental engineering at Virginia Tech University, has stated that the published instructions by EPA for collection of water samples for lead analysis were biased in the direction of underestimating the lead content of the water samples. He had spent years communicating this problem to EPA without a subsequent change in these instructions [6]. Dr. Edwards testified before Congress in spring 2016 that the Regional EPA Administrator was not alert to what was happening in Flint. Dr. Edwards also published papers previously bringing to the public attention the lead contamination of drinking water in Washington, DC. After Washington, DC made a change in its water disinfectant from chlorine to chloramine, residents were exposed to water with high levels of lead (140 ppb and above) from 2001 to 2004 [7,8]. This resulted in an increase of blood lead levels in young children (many from high-risk neighborhoods) of four times the amount that it was prior to the change in water disinfectant [7,8]. Dr. Edwards was a key player in ensuring that this issue was brought to light and those responsible parties were held accountable [9]. For comparison, the EPA standard for maximum contaminant level for lead in water is 15 ppb [5].

Regarding the Flint, Michigan water contamination incident, Dr. Mona Hanna-Attisha, a local pediatrician, performed a study looking at blood lead levels (BLLs) from Flint children from 2013 (before the water change) to 2015 (after the water change), assessing the percentage of BLLs over the Centers for Disease Control and Prevention (CDC) reference level of 5 µg/dL, reviewing water levels in Flint, and identifying geographical locations of blood and water levels using geospatial analysis. Her study demonstrated that the level of elevated blood lead levels (above 5 µg/dL) in a group of Flint children almost doubled between levels collected prior to the change in water source and afterwards; among children living in the area with highest water lead levels the percentage with elevated BLLs was approximately three times higher when compared to pre-diversion levels (4% versus 10.6%) [10]. These are extraordinary changes! (The specific blood lead levels or even range of BLLs was not reported in the article.) Unfortunately, many children in Flint already had multiple risk factors for lead poisoning, including “poor nutrition, concentrated poverty, and older housing stock” [10].

## 2. Elevated Blood Lead Levels in US Children and the Adverse Health Impacts and Costs of Exposure

Lead exposure in young children can lead to decrements in intelligence, development, behavior, attention, and other neurological functions. Two giants in childhood lead poisoning research and advocacy, Dr. Philip Landrigan and Dr. David Bellinger, summarize the adverse effects of lead very completely, yet succinctly: “Lead is a devastating poison. It damages children’s brains, erodes intelligence, diminishes creativity and the ability to weigh consequences and make good decisions, impairs language skills, shortens attention span, and predisposes to hyperactive and aggressive behavior. Lead exposure in early childhood is linked to later increased risk for dyslexia and school failure.” [11] Other articles and reports have also confirmed these adverse effects [12,13,14,15,16,17,18,19,20].

Therefore, it is important to determine the extent of the problem of elevated blood lead levels in U.S. children. Currently, based on data from the National Health and Nutrition Examination Survey (NHANES) from 2003 to 2012, 3.24% of children overall aged 1–5 years had a BLL > 5 µg/dL, compared with 7.8% of non-Hispanic Black (NHB) children [21]. Males had higher adjusted BLLs than females, and a higher poverty income ratio was associated with lower adjusted blood lead levels. Adjusted BLLs increased in renter-occupied (as opposed to owner-occupied) homes and with an increase in the numbers of smokers inside the home [21]. A previous analysis by Dixon et al. [22] of NHANES data from 1999 to 2004 found that BLLs were affected by the levels of lead in the floor and the condition of and surface type of the floor; that non-Hispanic Black children had higher BLLs than non-Hispanic white (NHW) children; that Mexican-born children had higher BLLs than those born in the U.S.; that houses built before 1940 were associated with children with higher BLLs; that children living in houses with a smoker had higher BLLs than those living with non-smokers; and that the odds of NHB children having BLLs > 5 µg/dL and > 10 µg/dL were more than double that of NHW children [21,22]. A recent report suggested that many children requiring blood lead testing due to Medicaid insurance status or state or city requirements for testing are not getting tested, and/or the results are not being properly followed up on [23].

The costs from lead poisoning are considerable, as are the cost savings for prevention of childhood lead poisoning. Attina and Trasande state that in the United States and Europe the lead-attributable economic costs have been estimated at $50.9 and $55 billion dollars, respectively [24]. Interestingly, they estimate a total cost of $977 billion of international dollars in low- and middle-income countries, with economic losses equal to $134.7 billion in Africa, $142.3 billion in Latin American and the Caribbean, and $699.9 billion in Asia, giving a total economic loss for these countries in the range of $728.6–$1,162.5 billion [24]. A previous analysis showed that each dollar invested in lead paint hazard control results in a return of $17–$221 or a net savings of $181–$269 billion for a specific cohort of children under six years of age as the benefits of BLL reduction would include categories such as health care, lifetime earnings, tax revenue, special education, attention deficit-hyperactivity disorder, and the direct costs of crime [25]. Another prior analysis estimated the economic benefits resulting from an historic lowering of children’s BLLs as measured by data from the National Health and Nutrition Examination Survey (NHANES) to be $110–$319 billion for each year’s cohort of 3.8 million two-year-old children, using a discounted lifetime earnings of $723,300 for each two-year-old child in 2000 dollars [26]. These estimated benefits were due to projected improvements in worker productivity due to increased intelligence quotient (I.Q.) points.

## 3. How the Flint Case and Other Examples Exhibit Environmental Injustice

Most affected by this egregious environmental disaster was a mostly poor and African-American population [27]. Some have speculated whether such an error in judgment might have occurred if a different population had been involved, and The New York Times uses the term racism in its editorial [27]. Another New York Times article talks of an analysis of emails from Governor Rick Snyder’s office that did not mention race but talked of costs involving Flint’s water supply, questioned scientific data regarding water contamination with lead, and mentioned uncertainties about the duties of state and local health officials [28]. It also mentions that some civil rights advocates were indicating that the Flint water crisis appeared to represent environmental racism [28]. The article goes on to discuss that the switch in water source was explicitly decided in favor of saving money for the financially unstable city of Flint, and that an emergency manager appointed by Gov. Snyder to carry out the running of the city was himself African-American [28]. One of Gov. Snyder’s key staff people sounded an alarm about the concern for lead in water, but the state health department responded back that the Flint water was safe [28].

The Flint Water Advisory Task Force, comprised of five experts in public health and water policy and convened by Governor Snyder, repeatedly stated in its findings that the Michigan Department of Environmental Quality (MDEQ) improperly and inaccurately described the Flint water as being safe, which unfortunately was then interpreted as accurate by other state agencies and city and county agencies [29]. The Task Force report also described the Flint water crisis as “a story of government failure, intransigence, unpreparedness, delay, inaction and environmental injustice”, and adds that the MDEQ failed in its responsibility to properly enforce drinking water regulations, while the Michigan Department of Health and Human Services (MDHHD) failed its mission to protect public health [29]. A recent article suggests these two agencies produced sampling data that were flawed, failed to provide accurate information to the Governor’s office, the EPA and the public, and did not respond appropriately when given information by environmental health and medical professionals [30]. The Task Force report also explains that state-appointed emergency managers replaced local decision-makers in Flint, thus removing “the checks and balances and public accountability that come with public decision-making” [29]. The group also credits the public and engaged Flint citizens with continuing to question government leadership (although the Task Force noted “callous and dismissive responses to citizens’ expressed concerns”), and the media for its investigative journalism of the crisis [29]. The Task Force’s conclusion was that “Flint water customers were needlessly and tragically exposed to toxic levels of lead and other hazards through mismanagement of their drinking water supply” [29]. The Flint Water Advisory Task Force suggests that the Michigan governor should issue an executive order to mandate training and guidance on environmental justice across all state agencies, with acknowledgement that the Flint crisis of water contamination is an example of environmental injustice which has fallen on a predominantly African-American community [29]. The Task Force issued 44 recommendations to remedy the results of the failure of proper governance and resultant lead poisoning [29,30].

Many have spoken out about this environmental injustice, including research scientists and clinicians [11,31,32,33] and public health professionals [34]. Even the EPA administrator, Gina McCarthy, is speaking about how Michigan evaded the EPA regarding the Flint water crisis and how this type of disaster cannot happen elsewhere [34,35]. Dr. Robert Bullard, dean of the School of Public Health at Texas Southern University, calls the Flint water crisis—leading to lead exposure and poisoning with long delays in addressing the problem—a classic case of environmental racism [36]. “Environmental racism is real…so real that even having the facts, having the documentation and having the information has never been enough to provide equal protection for people of color and poor people” [37]. He continues, “It takes longer for the response and it takes longer for the recovery in communities of color and low-income communities.” [37] He explains that regional EPA officials and state officials in Michigan responded first with a cover-up, “and then defensively—either trying to avoid responsibility or minimizing the extent of the damage”, as contrasted with handling of other environmental problems in predominantly white communities [37]. An example is then given of government officials on all levels helping to clean up a spill of coal ash in Roane County, Tennessee, in a mostly white community [37]. A Democrat who represents Flint, Michigan, Representative Dan Kildee, called race “the single greatest determinant of what happened in Flint” [28]. What is the solution? Dr. Bullard suggests that real solutions will result when communities previously left out of decision-making are offered a seat at the table [31]. In order to stop unequal protection from environmental hazards, Dr. Bullard has come up with five principles he suggests government adopt to further environmental justice: “guaranteeing the right to environmental protection, preventing harm before it occurs, shifting the burden of proof to the polluters, obviating proof of intent to discriminate, and redressing existing inequities” [37]. Charles Lee, another author writing about environmental justice who worked in the Office of Environmental Justice at EPA, quotes a definition of the environment as “the place where we live, where we work, and where we play” [38]. He goes on to state that “environmental justice must be the starting point for achieving healthy people, homes, and communities” [38]. Lastly, the Flint Water Task Force elaborates on its finding of environmental injustice in the Flint case. “Environmental justice or injustice, therefore, is not about intent. Rather, it is about process and results—fair treatment, equal protection, and meaningful participation in neutral forums that honor human dignity…The facts of the Flint water crisis lead us to the inescapable conclusion that this is a case of environmental injustice. Flint residents, who are majority Black or African American and among the most impoverished of any metropolitan area in the United States, did not enjoy the same degree of protection from environmental and health hazards as that provided to other communities” [29].

The reader is referred to several references [1,2,3] for a more detailed timeline of the specific events and actions that occurred in Flint. The Flint Water Task Force report also provides a summary of its findings and recommendations, giving greater details on the specific events and actions during the switch in water supply [29]. Regardless of the motivations behind the water supply mismanagement, we must improve governmental safeguards and public health surveillance to strive to avoid such needless exposures to environmental toxicants in the future.

Another recent disaster, involving contamination of local water supplies, was that of the contamination of the Animas River in southern Colorado and northern New Mexico by mine waste from the Gold King Mine, leading to excessively high levels of some toxic elements metals including lead, arsenic and cadmium (all of these being toxic metals) [39,40,41]. The river water was subsequently off limits for agricultural use and closed for recreational use [39,42]. The Navajo Nation has recently expressed how difficult and problematic this poisoning of their drinking water source has been to this tribe, and that they have not been adequately reimbursed for the adverse impacts to their water source and way of life [43,44]. The Native American Rights Fund states that a source of clean and abundant water is hard to come by for many Native tribes and peoples and that many face health and developmental risks from environmental problems such as surface and groundwater contamination, hazardous waste disposal, illegal dumping, and mining wastes, all of which can contribute to poor quality of water [45]. As the Flint, the Navajo Nation, and the Native American Rights group exposures highlight, poor and minority communities are unfortunately too often exposed to poisonous chemicals in their neighborhoods and on their tribal lands, leading to environmental injustice [44].

Not only has the incident in Flint brought to light the contamination of Flint’s water system, it raises issues about local water supplies to towns and cities, and particularly to child care centers and school systems, around the nation [46,47,48]. This has caused our nation to focus on investigating for lead contamination in water supplies in other cities, particularly in school systems, child care centers and other places occupied by children [49,50]. A Huffington Post article states that the Flint water crisis has provided a wake-up call to the country with the “discovery” of poisoned water in many communities in the U.S., and that our water infrastructure is outdated and deteriorating, and that water sampling procedures for lead are also “dangerously” outdated, as they allow for 10% of the population to be exposed to levels over the EPA maximum contaminant level [51]. Some cities have been cited for their exemplary actions in keeping their city water supplies free from lead contamination [52].

Historically, the scientists in the companies that put lead in gasoline and lead in paint became aware of the dangers of those specific lead exposures, but it took much time to finally remove lead from these products; many counties banned the use of lead-based paint in residential housing before the U.S. did [53,54]. One author states, “Flint’s tragedy is shedding light on a health issue that’s been lurking in U.S. households for what seems like forever. But that demands the question: Why has lead poisoning never really been treated like what it is—the longest-lasting childhood-health epidemic in U.S. history?” [55]. Bliss then goes on to describe how when in the 1950s, when “millions of children had had been chronically or acutely exposed (to lead)” and this had been linked to health problems, that “If the lead industry had stepped up then (or if it had been forced to by government)”, maybe lead poisoning would have been treated like any other major childhood disease—polio, for example. In the 1950s, “Fewer than sixty thousand new cases of polio per year created a near-panic among American parents and a national mobilization that led to vaccination campaigns that virtually wiped out the disease within a decade”, write Rosner and Markowitz [56]. “With lead poisoning, the industry and federal government could have mobilized together to systemically detoxify the nation’s lead-infested housing stock, and end the epidemic right there” [55]. Bliss then goes on to describe how “the industry’s powerful leaders diverted the attention of health officials away from their products, and toward class and race” by associating childhood lead poisoning with that of a child “with ‘ignorant’ parents living in ‘slums’” [55]. Bliss goes on to state that “lead poisoning in children can be eradicated…Today the cost of detoxifying the entire nation hovers around $1 trillion, says Rosner. Any federal effort to systematically identify and remove lead from infested households would be complex, decades-long, and require ongoing policy reform. ‘But it’s also saving a next generation of children,’ Rosner says. ‘You’re actually going to stop these kids from being poisoned. And isn’t that worth something?’” [56]. “And Rosner is a tiny bit hopeful. Amid national conversation about economic inequality, a housing crisis, and the value of black and Latino lives, the attention that Flint has brought to lead might usher in the country’s first comprehensive lead-poisoning prevention program” [56].

With the information about lead contamination in Flint and many cities around the country, one might wonder whether there is a dearth of information or recommendations about how to prevent and manage childhood lead poisoning. There is not. Many authors have weighed in on this question recently [11,12,13,15,17,19,57,58,59,60,61,62], some with very specific plans and ideas. Primary prevention of lead exposure has been particularly emphasized in almost all of them. Landrigan and Bellinger compel us to “map the sources of lead, get the lead out, and make sure there is no new lead” [11]. Jacobs and colleagues at the National Center for Healthy Housing have started a campaign for lead exposure detection and lead poisoning prevention based on these three principles: “find it, fix it, and fund it” [33]. Some call for revised standards for lead in air, house dust, soil and water [12,61,62,63]. The chief causes of lead exposure are nicely summarized by Levin and colleagues [64]. Unfortunately, childhood lead poisoning prevention is often deemed to be not important enough to work on, with other pressing medical and public health problems intervening; it is also complicated, complex and involves many stakeholders. Thus, the clinicians, government officials, and public health officials looking for a quick fix and a one-prescription answer to this medical problem are often disappointed and discouraged.

Concern about the neurotoxic effects of lead has been expanded now to include the neurotoxic effects of many more new chemicals out in use by the American public, including children. Children are exposed to chemicals in their everyday lives, as these are found in toys, children’s products, personal care items such as shampoos and skin creams, on foods in the form of pesticide residues, and in the air in the form of air pollutants. Some authors have weighed in on the need for more control of the manufacture and use of these toxicants and for more research into adverse health effects [31,65,66]. In 2015, a unique group of research scientists, clinicians, government representatives, and health care advocates met to form the Project TENDR (Targeting Environmental Neurodevelopmental Risks) which focuses on engendering action to prevent exposure of fetuses, infants and children to environmental toxicants [67]. The group has created a list of five chemical classes of neurotoxins which have adverse effects on brain development. The list includes lead, specific air pollutants, organophosphate pesticides, phthalates, and polybrominated diphenyl ethers (PBDEs), which are flame retardants. These were selected based on the degree of evidence for their adverse effects and the ability of this group and other scientists, clinicians, government officials, and advocates to work effectively to prevent exposures to these toxicants. Project TENDR has recently released a consensus statement with many signatures of both individuals and groups [67,68,69], as well as other articles on the project’s work [70]. Later this year, the group will release specific recommendations for prevention of exposure to the five chemical groups. The recent passage of the Frank R. Lautenberg Chemical Safety for the 21st Century Act has been a welcome revision and updating of the Toxic Substances Control Act promulgated by EPA in 1976 [71,72,73]. This is a step in right direction for better control of exposures to lead and other toxic chemicals in our environment.

## 4. Future Directions: How to Move Toward Environmental Justice

How do we remedy the situation in Flint, Michigan, and prevent future episodes similar to the Flint and Navajo Nation disasters? The Flint Water Task Force recommends that the MDHHS establish a Flint Toxic Exposure Registry to follow-up on the children and adults who were residing in Flint from April 2014 until the present, and carry out more aggressive clinical and public health follow-up for all children with elevated BLLs in the state [29]. It also recommends that routine lead screening and appropriate follow-up occur in the children’s medical homes (with the primary care provider) [29]. Additionally, the Task Force recommends that the Genesee County Health Department improve follow-up of health concerns in cooperation with the MDHHS and City of Flint “to effect timely, comprehensive, and coordinated activity and ensure the best health outcomes for children and adults affected” [29]. Dr. Hanna-Attisha has established the Flint Child Health and Development Fund which will support children and their families to obtain the optimum health and development outcomes, early childhood education, access to a pediatric medical home, improved nutrition and integrated social services [6]. The Michigan State University (MSU)/Hurley Pediatric Public Health Initiative will assess, monitor, and intervene to increase children’s readiness to succeed in school by providing the above services, along with stimulating environments and parenting education [6]. This type of close follow-up has been recommended under the Flint Recovery and Remediation section of the Flint Water Task Force, as well as a recommendation to establish a dedicated subsidiary fund in the Michigan Health Endowment Fund for funding health-related services for Flint residents [29]. Therefore, local efforts will be taken to counteract the negative consequences of exposure to lead for Flint’s children. Several recent publications support the positive effects that enriched home environments can have on cognition and behavior in both human and animal studies [74,75,76].

Secondly, government agencies at the federal, state and local level and municipal authorities will need to improve their performance to ensure environmental justice, rather than contribute toward environmental injustice. This was mandated in an Executive Order by President William Clinton which requires all federal agencies to take action to ensure environmental justice [77]. The American Academy of Pediatrics provides a good starting point regarding childhood lead exposure prevention with their recommendation that “The US EPA and HUD should review their protocols for identifying and mitigating residential lead hazards (e.g., lead-based paint, dust, and soil) and lead-contaminated water from lead service lines or lead solder and revise downward the allowable levels of lead in house dust, soil, paint, and water to conform with the recognition that there are no safe levels of lead” [12]. They also give many other recommendations for government, as well as for pediatricians and other health care providers, for reducing and preventing children’s exposure to lead. Other groups, authors and reports have weighed in on what needs to be fixed and carried out, as indicated earlier in this article. As Bellinger puts it, “We know where the lead is, how people are exposed, and how it damages health. What we lack is the political will to do what should be done” [32].

Looking at the Flint case specifically, why was the water supply switched in Flint? The evidence seems to point to financial reasons for this. In Flint, state officials decided to save money without concern for providing environmental protections for a community at well-established increased risk. This is clear injustice in environmental protection to a low income and minority community. Why weren’t the corrosion control measures implemented? The Flint Water Task Force implicates various leadership groups, including the MDEQ, MDHHS, Michigan’s Governor’s Office, State-appointed Emergency Managers, the EPA, and City of Flint, although the MDEQ and EPA seem to share most culpability [29].

## 5. Conclusions

In short, this crisis was the result of failures on every level. We have presented various comments about how environmental racism and injustice played into this situation. Why were the concerns and complaints about water quality from a mostly African-American community not addressed? The facts presented demonstrate that environmental injustice is the major and underlying factor involved in the events in Flint. Having a state-appointed emergency manager in charge took away the normal communication the City of Flint might have had with its residents and constituents. The Flint Water Task Force has a list of 44 recommendations, mostly directed at the various agencies and offices involved, for improving the situation and preventing further problems [29]. Much of this involves recommending that these entities seek and follow expert advice, whether on water treatment techniques or protecting the public’s health [29]. It is also imperative to rebuild relationships with Flint’s community and respond to community needs in order to make real and lasting change. Perhaps putting the Flint situation under a microscopic analysis may prevent future episodes of such environmental injustice.

We must do a better job at moving forward and preventing environmental injustice; our future work is cut out for us.
